# Annotations

**Published:** 1887-02-19

**Authors:** 


### ANNOTATIONS.

**Waking up at the Dorset County Hospital.**

THE Annual Meeting of the Governors of the Dorset County Hospital, just held, was enlivened by some rather sharp discussion. A governor complained that he and his fellows were asked to vote approval of the annual report without having had an opportunity of previously making themselves acquainted with it. It was usual, he said, at other institutions, to send round to governors a printed copy of the report some days before the meeting. Then, of course, each member could, if he chose, study it in detail, and might be prepared to offer an intelligent opinion. But to ask members to vote upon a series of official figures at a moment's notice was decidedly unbusiness-like—not to say absurd. Notwithstanding that the governor in question had only received his copy of the report on entering the meeting, he sharply criticised several items of expenditure. Although his objections were not sustained, a resolution was passed to the effect that in future each governor should have sent to him a printed copy of the annual report of the institution at least a week before the annual meeting. It is to be hoped that there are no other Boards of Governors who are called upon to vote approval of the reports of their hospitals in the same way. If there be they should at once insist upon such a reformation as that which has taken place at the Dorset Hospital. Nothing can be more injurious to charitable institutions than leaving the accounts and expenditure entirely in the hands of officials. In every case the governors should make themselves acquainted with the details of management, and give no votes of approval or otherwise without a full knowledge of the facts. A motion to exclude members of the medical staff from the committee of management was decisively and very properly rejected.

**A Third Hospital for Glasgow.**

The cannie folks of the West of Scotland are thinking of taking up a new enterprise of some magnitude. They have already two large general hospitals, one of which is hardly a dozen years old. A third is in contemplation, if it has not already reached a more advanced stage. To people familiar with what is almost, if not quite, the second city in the kingdom, the proposal will occasion no surprise. There is a vast working-class population in Glasgow, of which a large proportion is resident on the South side, where the new hospital is intended to be built. Not only is there a large working-class contingent on that side, but its denizens are to a considerable extent Irish. That means that they are not so well off or so provident as the ordinary working population of Scottish towns, and therefore, when sickness comes upon them, they have no resource but the hospital or the poor-house. On this account it is satisfactory to find that hospital provision is in contemplation. The South-side doctors, too, will be glad to have a hospital brought nearer to them. Medical practice is apt to degenerate where the stimulus of such an institution is wanting. We hope the promoters may receive abundant support in what is not only a good, but a much-needed work.

**Nurses at Play.**

The English are not a merry people, although our country is called "Merrie England." Some people seem to be compelled by the nature of their avocations to a kind of perpetual solemnity. Among these may be named undertakers, Evangelical clergymen, and hospital nurses. An undertaker waltzing or playing the fiddle, would be as unnatural and incongruous to conventional ideas as a bullock saddled for the hunting-field. Nevertheless, one can imagine it possible that an undertaker might wish to get out of the atmosphere of perpetual crape, and even take pleasure in so frivolous an occupation as a Schottische or a Scotch reel. Why not? The poor hospital nurse must sometimes wish for a little change. Does anybody ever invite her out? Does she sometimes spend an evening among young people, where the song, and the game, and the merry dance make her for awhile remember that the whole world is not a hospital, and all the men and women are not patients? It is to be feared that such innocent delights seldom fall to her lot. The more is the pity. People should not be selfish in their grief and sorrow. Nurses are women, and often young women; and if they give themselves up to the service and comfort of the sick, the well should try to make their tedious and melancholy duties as endurable as possible by the frequent infusion of a little innocent pleasure into their lives.

